# Atypical hemolytic-uremic syndrome associated with antiphospholipid antibodies and antiphospholipid syndrome; a novel presentation

**DOI:** 10.1186/1546-0096-12-S1-P363

**Published:** 2014-09-17

**Authors:** Reza Shiari, Vadood Javadi Parvaneh, Reza Dalirani, Shirin Farivar, M Reza Shiva

**Affiliations:** 1 Pediatric Rheumatology, Shahid Beheshti University Of Medical Sciences, Tehran, Iran, Islamic Republic Of; 2 Pediatric Nephrology, Shahid Beheshti University Of Medical Sciences, Tehran, Iran, Islamic Republic Of; 3 Genetics, Shahid Beheshti University, Tehran, Iran, Islamic Republic Of

## Introduction

Atypical hemolytic-uremic syndrome (HUS) is defined by the presence of microangiopathic hemolytic anemia, acute renal failure, and thrombocytopenia without a diarrheal prodrome. It is responsible for only ten percent of cases in children. The role of genetic deficiencies of complement regulation, Von Willebrand factor cleaving protease (ADAMTS 13), and intracellular defects of vitamin B12 metabolism has been known in pathogenesis of disease. Antiphospholipid antibodies (aPLs) are autoantibodies against negatively charged phospholipids or phospholipid binding plasma proteins. It is clear that their presence is associated with thrombosis, pregnancy morbidity, hematologic, skin, neurological conditions, and microangiopathy.

## Objectives

To our knowledge, there are only two pediatric case series that showed a high frequency of anticardiolipin antibodies in children with typical HUS. Microangiopathic antiphospholipid-associated syndrome (MAPS) was also described in a child with atypical HUS.

Herein we reported a 5.5-year-old boy who was presented with atypical HUS associated with antiphospholipid antibodies and antiphospholipid syndrome in his father.

## Methods

A 5.5-year-old boy from Azerbaijan was referred to Mofid Children's Hospital, because of edema, hypertension, anemia and acute renal failure. He was healthy until 4 weeks before admission while he had fever and sore throat. Then generalized edema, echymosis, gross hematuria and sever paleness occurred. The history of diarrhea was negative. On presentation, he was ill, pale but afebrile.

## Results

His laboratory data on admission were as following: white blood cell (WBC): 18800/µL, hemoglobin (Hb): 11g/dl, lactate dehydrogenase (LDH): 6286 Iu/ml, creatin phosphokinase (CK): 1039 u/L, Erythrocyte sedimentation rate (ESR): 40 hr, C-reactive protein (CRP): 11 mg/L, blood urea nitrogen (BUN): 98 mg/dl, creatinin: 3.7 mg/dl, uric acid: 16.8 mg/dl, Aspartate transaminase (AST): 298 Iu/L , Alanine transaminase (ALT): 37Iu/L. His urinalysis showed hematuria (many RBC) and proteinuria (2+). He had low level of C_3_: 30 mg/dl (normal range: 90-180) and his C4 level was 11(10-40) mg/dl.

## Conclusion

On the basis of microangiopathic hemolytic anemia, thrombocytopenia and acute renal failure, he was diagnosed as having atypical HUS. To our knowledge, in the literature, there is no report of this association between aHUS , aPS and aPLs as predisposing factor.

## Disclosure of interest

None declared

